# Mechanical feedback cooling assisted by optical cavity cooling of the thermal vibration of a microcantilever

**DOI:** 10.1038/s41598-019-55496-x

**Published:** 2019-12-13

**Authors:** Y. Kawamura

**Affiliations:** Department of Intelligent Mechanical Engineering, Fukuoka Institute of Technology, 3-30-1 Wajirohigashi, Higashi-ku, Fukuoka, 811-0295 Japan

**Keywords:** Nanoscience and technology, Optics and photonics, Physics

## Abstract

This study describes a new two-step process to cool the thermal vibration of microcantilevers. The process combines active mechanical feedback cooling and optical cavity cooling. A micro-Fabry–Perot interferometer, built in-house, is set atop a microcantilever to measure the vibration amplitude, the high optical power density of which induces cavity cooling in the optical cavity. Using a two-step cooling procedure, the equivalent temperature of the thermal vibration of a microcantilever is lowered from room temperature to the theoretical cooling limit of 0.063 K, a much lower temperature than that achieved via simple cavity cooling (18 K), and then by mechanical feedback cooling (0.135 K) obtained for the same type of microcantilevers in previous studies. This experimental demonstration showcases a new type of cooling process of the amplitude of thermal vibration for micro-mechanical resonators to a lower temperature and does not need additional cooling using a conventional cryogenic refrigerator.

## Introduction

There have been several proposals and attempts^1–4^ to decrease, as much as possible, the amplitude of thermal vibration of micro-resonators, ideally down to the quantum limit^5–7^. One such proposal uses a silicon microcantilever. Methods for damping thermal vibration fall into three categories. The first is direct cryogenic cooling of the microcantilever, in which all of the vibration modes are equally cooled, but the experimental system for this is very large and complex^8–10^. The second is active mechanical feedback damping of the vibration amplitude^11–14^, in which only selected vibration modes are cooled down^15^. The feedback control system becomes sensitive to external vibration, but is relatively small. The third is a self-cooling method called cavity damping by which the amplitude of a thermal vibration is damped using bolometric effects^16–18^ or quantum effects^19^. The system for this is simple and small.

There have been a few trials with two-step cooling processes using initially a cryogenic means and then mechanical feedback that have lowered the temperature of cantilevers^11^. However, there has been no study of two-step cooling processes using feedback and then cavity cooling, as far as we know. This study demonstrates the latter two-step cooling using simultaneously mechanical-feedback and optical-cavity cooling.

## Methods

**Figure 1:**
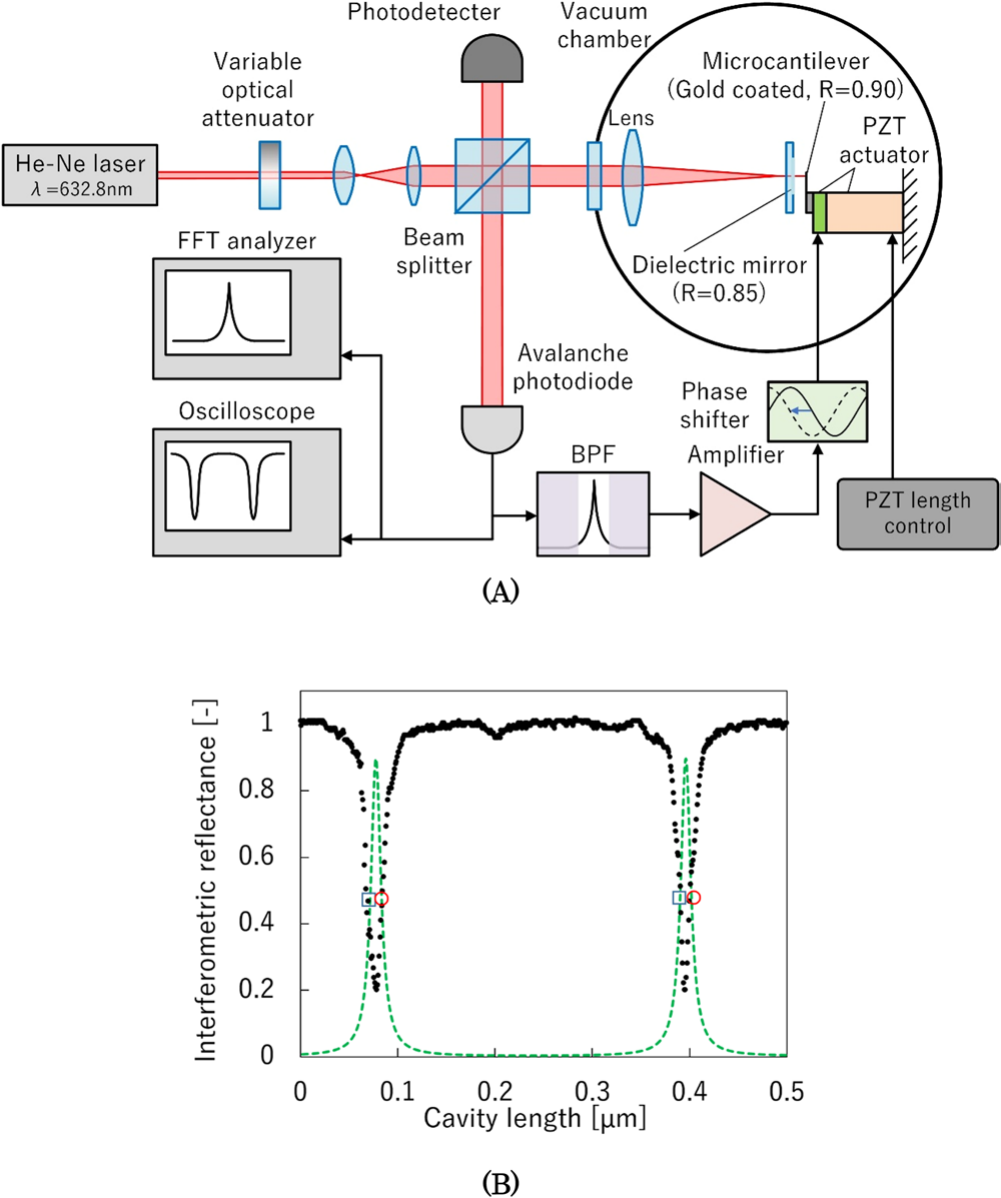
(**A**) Experimental setup for two-step damping of the thermal vibration using a positive mechanical feedback cooling and passive cavity cooling. (**B**) Typical reflection signal of the micro-Fabry–Perot interferometer as a function of cavity length. Green dotted line is the relative optical power in the cavity. Red open circles mark the operation points of “cavity heating”, and blue open squares mark the operation points of “cavity cooling”.

The experimental setup for the hybrid cooling of the vibration amplitude of a silicon microcantilever [Fig. 1(A)] consists of a mechanical cantilever for an atomic force microscope that is commercially available with a length, width, and thickness of 240 µm, 40 µm, and about 2 µm, respectively^20^. The catalog value of the spring constant *k* is given as approximately 2 N/m. The natural frequencies of the microlever are *f*_0_ = 58.4 kHz and 76.2 kHz. One side of each cantilever was gold-coated via ion sputtering to construct a Fabry–Perot interferometer with a reflectance of about 90%^21^. The Fabry–Perot interferometer has a flat dielectric paring mirror with a reflectance of 92%. The finesse of the interferometer was measured to be 25^21^. The cantilever was mounted on a single layer piezoelectric transducer (PZT) for precise movements. The system was placed in a vacuum chamber evacuated down to 5 × 10^−3^ Pa using an oil diffusion vacuum pump.

The vibration amplitude of the silicon microlever was measured by the Fabry–Perot interferometer. A He–Ne laser producing a beam of wavelength 632.8 nm was used as a light source for the interferometer. Using an optical attenuator, the laser power of the interferometer can be varied from 0 to 850 µW. The output signal of the interferometer was passed through a band-pass filter with a bandwidth of 13 kHz. The signal phase was delayed by 90° to dampen the vibration amplitude^12^. The loop gain *g* of the feedback control was changed by altering the amplification ratio of the drive voltage of the PZT. Being the gain of the feedback loop, *g* is defined as the ratio of the movement of the PZT to that of the cantilever. The entire experimental system was placed on an anti-vibration table, and the interferometer system and the microcantilever were mounted on a plate supported by soft rubber blocks in the vacuum chamber to isolate both from further vibration. The output signal from the photodetector was recorded and processed using a fast Fourier transform spectral analyzer. The quality factor *Q* of the resonance was measured to be around 7000. When also measured for the same silicon cantilever without gold coating, the *Q* value was up around 1.0 × 10^5^ ^12^, which means that the gold coating increased the damping factor and decreased *Q*.

Cavity cooling is considered to be induced by the delay of thermal conduction in the gold-coated silicon cantilever and is a type of passive thermal feedback damping^16^. Therefore, cavity cooling in this experimental system has occurred spontaneously.

Typical reflection signals of the Fabry–Perot interferometer as a function of the gap length of the optical cavity [Fig. 1(B)] show an inverted reflection signal (green dotted line), which is just the relative optical power in the cavity. The operation point of the interferometer was automatically kept on the cooling points of the cavity by changing the cavity gap length using a PZT (length control). These cavity cooling points, indicated by open blue squares in Fig. 1(B), were found in this experimental setup by adjusting the optical alignment of the Fabry–Perot interferometer; see Supplementary Materials.

**Figure 2:**
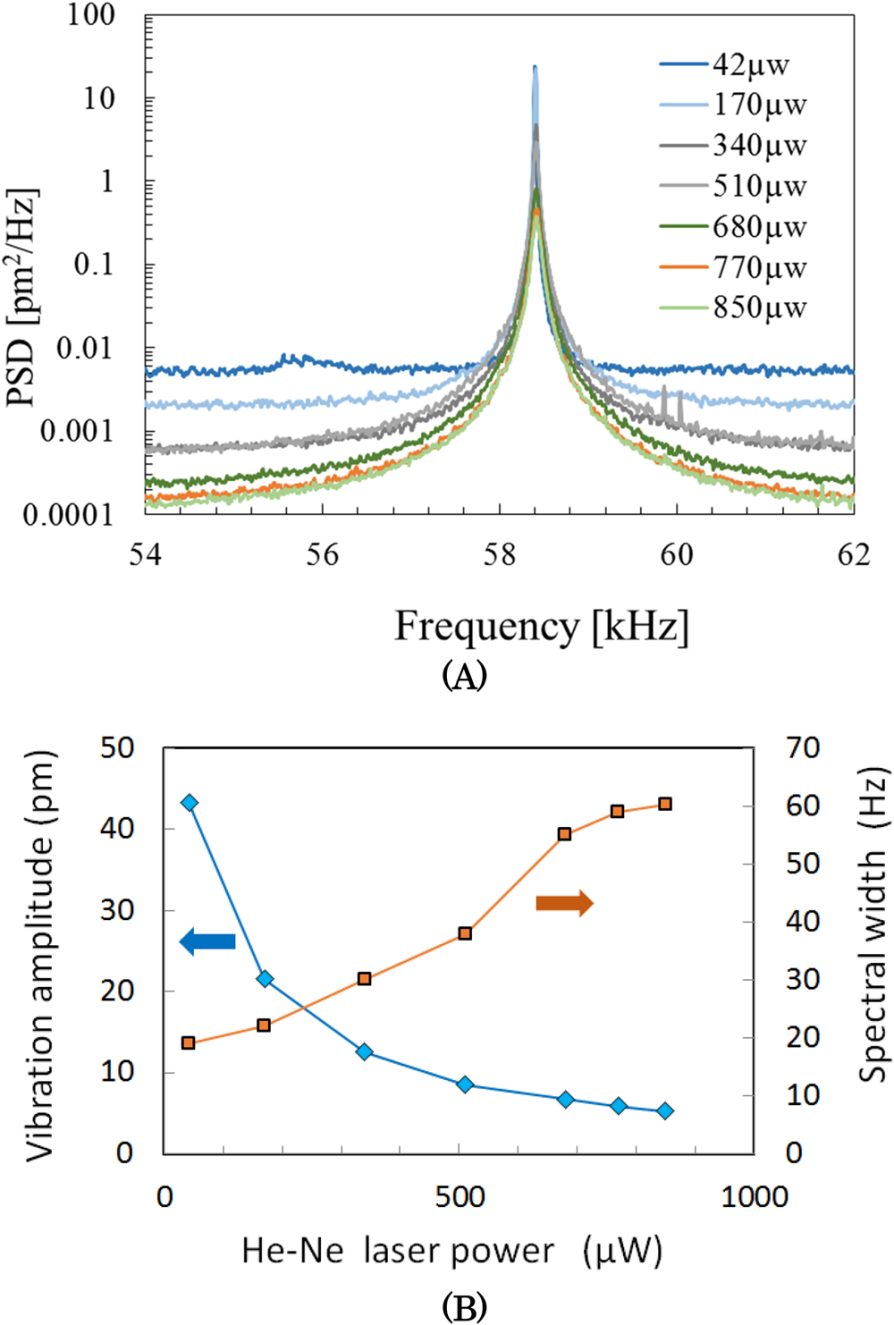
(**A**) Changes in the power spectrum density of the microcantilever induced by optical cavity cooling for various He–Ne laser power, without mechanical feedback cooling. (**B**) Vibration amplitude and spectral width as a function of He–Ne laser power, calculated using the data in (**A**).

For various He–Ne laser powers in the cavity-cooling mode, without mechanical feedback cooling, the vibration power spectral densities (PSDs) of the microcantilever were obtained [Fig. 2(A)] from which the vibration amplitude and spectral width were calculated [Fig. 2(B)]. The behaviors of the spectrum width and vibration amplitude are the same as those obtained from mechanical feedback cooling and obtained with increasing mechanical gain. Therefore, the laser power in the former case is considered to correspond to the mechanical feedback gain in the latter. The vibration amplitude is obtained by integrating the PSD with respect to frequency, and taking the square root. We see an increase in width of the PSD, as well as a decrease in vibration amplitude with increasing laser power. The behaviors of these two experimental parameters (spectrum width and peak value) are the same as those for mechanical feedback cooling, both of which were obtained with increasing mechanical gain. Therefore, the laser power in the former case is considered to correspond to the mechanical feedback gain in the latter. The amplitude was calculated to be 5.3 pm, which corresponds to an equivalent temperature of 4.0 K.

When the operational points were set [Fig. 1(B), red open circles], cavity heating occurred and the amplitude increased to the saturation level of the measurement system (about 10 nm).

**Figure 3:**
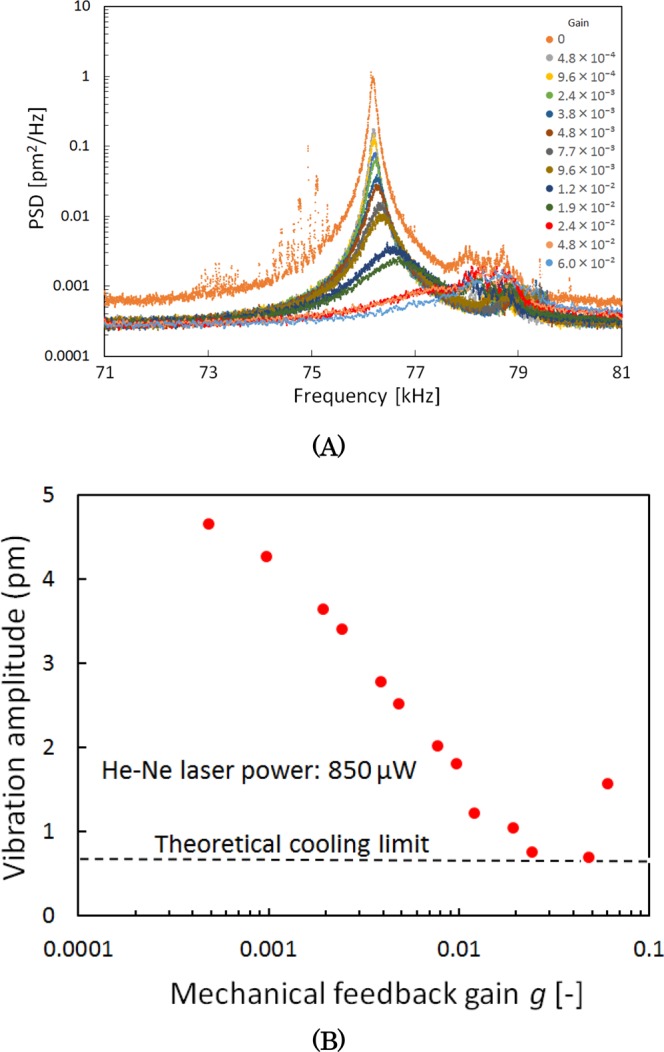
(**A**) Power spectral density of the vibration amplitude for various mechanical feedback gains in the two-step cooling experiment. He–Ne laser power is 850 µW. (**B**) Vibration amplitude obtained by a two-step operation of cavity cooling and conventional mechanical feedback cooling as a function of mechanical feedback gain. Dotted black line marks the theoretical limit to attain the minimum vibration amplitude of 0. 66 pm^22^.

Figure 3(A) shows the PSD of the vibration amplitude for various mechanical feedback gains in the two-step cooling experiment. The power of the He–Ne laser was set to 850 µW to maximize the effect of optical cooling. Figure 3(B) shows the vibration amplitude, obtained by integrating the PSD over the spectrum. As the feedback gain increased, the amplitude of the thermal vibration decreased, and the minimum amplitude of about 0.7 pm was obtained for *g* ≅ 0.03.

For higher values of *g* (not included in this figure), an oscillation of the feedback system appeared with an amplitude reaching the saturation level of the measurement system (about 10 nm)

The theoretical thermal vibration amplitude was calculated to be 4.5 × 10^−11^ m (45 pm), assuming that the energy *k*_B_*T/*2 is distributed as an average potential energy; here, *k*_B_ is Boltzmann’s constant, and *T* the ambient temperature (300 K)^12^.

The theoretical minimum attainable amplitude of 0.66 pm [Fig. 3(B); dotted black line] was obtained via the effective cooling temperature *T*_eff,min_ given by^11,22^

$${T}_{{\rm{eff}},{\rm{\min }}}=\sqrt{\frac{k{\omega }_{0}T}{{k}_{{\rm{B}}}Q}{S}_{n}},$$

where *k* denotes the spring constant of the cantilever, *T* the initial effective temperature of the resonators for *g* = 0, *ω*_0_ the natural angular frequency of the cantilever, and *S*_*n*_ the PSDs of the floor noise. Here, *T* equals 4.0 K, *ω*_0_ is 2π × 7.62 × 10^4^ rad/sec, *S*_*n*_ is 1 × 10^−4^ pm^2^/Hz, and *Q* is 7000. Therefore, *T*_eff,min_ evaluates to 0.063 K, corresponding to an amplitude *x*_eff,min_ = 0.66 pm.

In conclusion, a new two-step process was demonstrated for cooling the thermal vibration of a microcantilever. This process involves optical cavity cooling and mechanical feedback control applied simultaneously. The experimentally obtained minimum temperature is in good agreement with the theoretical cooling limit, which was calculated using the initial effective temperature and the floor noise obtained through cavity cooling.

## Data availability

The author declares that all the relevant data supporting the findings of this study are available within the article or from the corresponding author upon request.

## Supplementary information

Supplementary Information
