# Supplementary material for: Mechanical feedback cooling assisted by optical cavity cooling of the thermal vibration of a microcantilever
Source: Sci Rep. 2019 Dec 13;9:19094. doi: 10.1038/s41598-019-55496-x (PMC6910902; doi:10.1038/s41598-019-55496-x)
Supplement: Supplementary file 1 — Supplementary Information [file 41598_2019_55496_MOESM1_ESM.docx]

**Supplementary materials**

Mechanical feedback cooling assisted by optical cavity cooling of the thermal vibration of a microcantilever

(Observation of the cavity heating and cooling through the optical alignment of the Fabry–Perot (PF) interferometer.）

Y. Kawamura

Department of Intelligent Mechanical Engineering, Fukuoka Institute of Technology, 3-30-1 Wajirohigashi, Higashi-ku, Fukuoka 811-0295, Japan ([kawamura@fit.ac.jp](mailto:kawamura@fit.ac.jp))

The yellow and blue traces in Fig. S1 show respectively the oscilloscope trace of the Fabry–Perot interference (FPI) signal and the applied voltage to the piezoelectric transducer (PZT). The gap distance of the micro-FP interferometer is able to be continuously changed to find its best optical alignment position. The stable symmetrical dip profiles of the FPI were observed for He–Ne laser power lower than a certain threshold value. When the laser power was larger than this threshold, a spikey signal of unknown source was observed on one side of the dips, where the applied voltage to the PZT was decreasing, specifically, when the gap length of the FPI was decreasing. These spikey signals are believed to be from optical cavity heating [S1]. Therefore, with that assumption, cavity cooling must have occurred on the other side of the dip profile of the FPI signal.


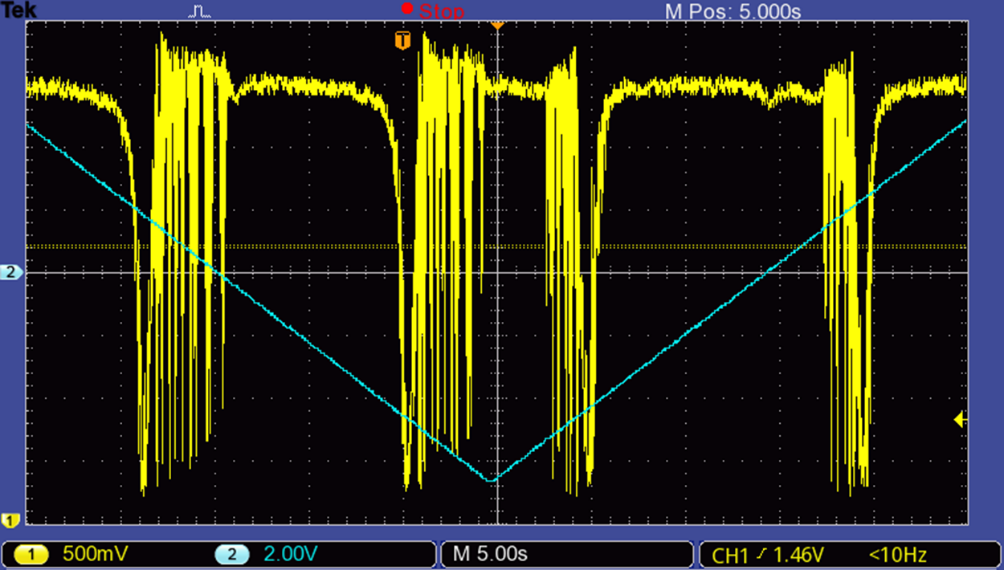


Fig. S1. Oscilloscope trace of the interference signal of the Fabry–Perot interferometer (yellow line) and applied voltage to the PZT (blue line).

**Reference**

S1 C. H. Metzger and K. Karrai, Cavity cooling of a microlever, Nature, **432**, 1002-1005 (2004)
